# The Hsp40 co-chaperone DNAJC7 modifies polyglutamine but not polyglycine aggregation

**DOI:** 10.1101/2025.08.10.669490

**Published:** 2025-08-12

**Authors:** Biswarathan Ramani, Kean Ehsani, Martin Kampmann

**Affiliations:** 1 Department of Pathology, University of California, San Francisco, San Francisco, CA, USA; 2 Institute for Neurodegenerative Diseases; Weill Institute for Neurosciences, University of California, San Francisco, San Francisco, CA, USA; 3 Department of Biochemistry and Biophysics, University of California, San Francisco, San Francisco, CA, USA

## Abstract

Polyglutamine (polyQ) diseases, including Huntington’s disease and several spinocerebellar ataxias, are caused by abnormally expanded CAG nucleotide repeats, which encode aggregation-prone polyQ tracts. Substantial prior evidence supports a pathogenic role for polyQ protein misfolding and aggregation, with molecular chaperones showing promise in suppressing disease phenotypes in cellular and animal models. In this study, we developed a FRET-based reporter system that models polyQ aggregation in human cells and used it to perform a high-throughput CRISPR interference screen targeting all known molecular chaperones. This screen identified as a strong suppressor of polyQ aggregation the Hsp40 co-chaperone DNAJC7, which has previously been shown to modify aggregation of other disease proteins (tau and TDP-43) and has mutations causative for amyotrophic lateral sclerosis. We validated this phenotype and further established a physical interaction between DNAJC7 and polyQ-expanded protein. In contrast, DNAJC7 did not modify aggregation of polyglycine (polyG) in a FRET-based model of neuronal intranuclear inclusion disease. In addition to establishing new inducible, scalable cellular models for polyQ and polyG aggregation, this work expands the role of DNAJC7 in regulating folding of disease-associated proteins.

## Introduction

An increasing number of neurodegenerative diseases are now recognized to be caused by nucleotide repeat expansions that are in protein-coding regions. Among these are the polyglutamine (polyQ) disorders caused by CAG repeat expansions, including Huntington’s disease (HD), spinocerebellar ataxias (SCA1, 2, 3, 6, 7, 17, and 51), spinobulbar muscular atrophy, and dentatorubropallidoluysian atrophy [1,2]. Similarly, diseases such as neuronal intranuclear inclusion disease (NIID) and fragile X-associated tremor/ataxia syndrome have more recently been linked to GGC repeat expansions that encode polyglycine (polyG) tracts. A common neuropathologic hallmark of these disorders is the accumulation of misfolded, aggregated proteins in the nucleus of neurons, often forming discrete intranuclear inclusions that immunostain with the autophagy adapter protein p62 [3–5].

Strong evidence implicates misfolded protein aggregates as pathogenic drivers in neurodegeneration. In both model systems and human patients, longer CAG repeats correlate with increased protein aggregation and more severe clinical phenotypes. Moreover, molecular chaperones, proteins that assist in folding and refolding of misfolded proteins, have been shown to suppress polyQ aggregation and mitigate toxicity in disease models [6–10]. These findings underscore the importance of the molecular chaperone network (i.e. the chaperome) and the broader proteostasis network in disease pathogenesis, highlighting their potential as therapeutic targets [11,12]. Importantly, molecular chaperones and co-chaperones represent a large and very diverse group of over 300 proteins, with only a subset of chaperones examined as possible modifiers of polyQ aggregation.

Here, we report the development of a Förster Resonance Energy Transfer (FRET)-based human cellular model of polyQ disease that exhibits spontaneous nuclear aggregation along with recapitulating other key features resembling human neuropathology. Moreover, the FRET readout enables high-throughput assessment of aggregation status in individual cells via flow cytometry. Using this model, we show the results of an unbiased CRISPR interference (CRISPRi) screen targeting all known molecular chaperones and co-chaperones, revealing DNAJC7 as a strong modifier of polyQ aggregation. We also developed a comparable FRET-based model for polyG nuclear aggregation, which exhibits similar microscopic features but is notably unaffected by DNAJC7 perturbation. Along with expanding the role of DNAJC7 in targeting pathogenic proteins, this study demonstrates the utility of FRET-based CRISPRi screening for uncovering regulators of protein aggregation in repeat expansion disorders.

## Materials and methods

### Animals

All mice were maintained according to the National Institutes of Health guidelines and all procedures used in this study were approved by the UCSF Institutional Animal Care and Use Committee. Mice were housed on a 12-h light/dark cycle at 22–25 °C, 50–60% humidity, and had food and water provided ad libitum. The mice used in this study were R6/1 mouse model of HD (B6.Cg-Tg(HDexon1)61Gpb/J, RRID:IMSR_JAX:006471), which are crossed onto a homozygous for Cre-inducible CRISPR interference machinery (B6;129S6-Gt(ROSA)26Sortm2(CAG-cas9*/ZNF10*)Gers/J, RRID: IMSR_JAX:033066). For genotyping HD mice, genomic DNA was purified from ear punches following the manufacturer’s instructions (New England Biolabs, T3010S). 1 μL of the eluted DNA was used for PCR amplification using primers and cycling conditions detailed in Supplementary Table 1.

### Plasmid construction and lentivirus packaging

A list of all plasmids used in this study with information on their main expression elements is provided in Supplementary Table 1. A map of each plasmid in a GenBank file format is provided in a Supplementary File. The FRET-based aggregation reporters were constructed on a lentiviral pLEX-TetOne backbone kindly provided by Michael Ward (National Institutes of Health) for doxycycline-inducible expression under a Tet-ON 3G promoter. C-terminal ataxin-3 (amino acid 257 to the C-terminus) containing a 79 consecutive glutamine stretch was cloned by PCR from pcDNA3-eGFP-Ataxin3Q84 [13] (Addgene #22123, a gift from Henry Paulson). Huntingtin exon 1 (HTTex1) with Q25 and Q72 repeats were cloned from pGW1-HTTN586 constructs [14], kindly provided by Steven Finkbeiner (Gladstone Institutes). The upstream ORF of the *NOTCH2NLC* encoding 100 glycines [15] (Addgene #224356, a gift from Nicolas Charlet-Berguerand) was cloned by restriction digestion into the pLEX-TetOne backbone. mNeonGreen, mScarlet, and eGFP were cloned by PCR. A 2 × c-myc nuclear localization signal and the mTagBFP2 (BFP) sequences were PCR amplified from pMK1334 [16]. The cDNA for DNAJC7 was kindly provided by Dr. Jason Gestwicki (UCSF). BFP and BFP fused to DNAJC7 were cloned by PCR into pMK1200 [17], a lentiviral backbone with an EF1α promoter.

Transient transfection experiments that used GFP-HTTex1-Q72 and 3×FLAG-tagged constructs were built on an AAV backbone with an EF1α promoter [18] (Addgene #55636, a gift from Karl Deisseroth). The 3×FLAG tag was inserted by annealing synthesized oligonucleotides (Integrated DNA Technologies).

The CRISPRi molecular chaperone sgRNA library used in this study was described previously [19]. Individual sgRNAs for validation studies were selected and cloned into pMK1334 lentiviral plasmid backbone between *BstXI* and *BlpI* by annealing and ligating annealed oligonucleotides. The sgRNA protospacer sequences are provided in Supplementary Table 1.

Lentiviral packaging was performed as previously described [20]. Briefly, HEK293T cells were seeded in complete DMEM to reach approximately 70% confluency the following day. For transfection, third-generation lentiviral packaging plasmids (pRSV, pMDL, and pVSV-G) were mixed at a 1:1:1 mass ratio (lentiviral pack-mix) and combined with an equal mass of the transfer plasmid. The DNA mixture was diluted in Opti-MEM and complexed with polyethylenimine (PEI; Polysciences, 23966) at a 3:1 PEI:DNA mass ratio. After 15 min of incubation at room temperature, the transfection mixture was added dropwise to the cells. Conditioned media was collected 48 h post-transfection and filter-sterilized using a Millex-GV syringe filter unit (Millipore, SLGV033RB). Lentivirus was then precipitated using the Alstem Lentivirus Concentration Kit (VC100) according to the manufacturer’s instructions and resuspended in 1× DPBS (Sigma-Aldrich, D8537).

For pooled sgRNA library packaging, 15 μg of sgRNA library DNA and 15 μg lentiviral pack-mix) were transfected into HEK293T cells plated in a 15 cm plate format. The precipitated virus was resuspended in 5 mL of 1× DPBS. For individual sgRNAs cloned into the pMK1334 backbone, 1 μg of the transfer DNA and 1 μg lentiviral pack-mix were transfected onto HEK293T cells plated in a 6-well (35mm) plate format. The resulting virus was resuspended in 200 μL of 1× DPBS.

### Cell culture and cell line generation

All cells were maintained in a tissue culture incubator (37 °C, 5% CO2) and checked regularly for mycoplasma contamination. HEK293T cells were cultured in DMEM supplemented with 10% fetal bovine serum (Seradigm 89510–186), Penicillin-streptomycin (Gibco, 15140122), and L-glutamine (Life Technologies, 25030081).

The doxycycline-inducible NLS-FRET-Q79 and NLS-FRET-G100 cell lines were made on the “cXG284” HEK293T cell line that has stably integrated CRISPRi machinery (dCas9-BFP-KRAB) in the CLYBL locus [21]. The cells were transduced with lentivirus and after at least 72 h and in the presence of 2 ng/ml doxycycline, the cells were dissociated and sorted for those expressing both mNeonGreen and mScarlet using a BD FACSAria FusionCell Sorter. Monoclonal lines were obtained by plating cells at limiting dilution, followed by screening for cells that showed the highest frequency of inclusions and FRET signal. The GFP-HTTex1-Q72 monoclonal cell line was generated similarly but used HEK293T cells expressing dCas9-BFP-KRAB introduced by random integration of lentivirus.

CRISPRi knockdown of individual genes was performed by transducing the indicated cell lines with pMK1334 lentivirus. To permit sufficient time for gene knockdown, doxycycline treatment to begin FRET reporter expression was initiated at least 5 days after transduction.

### Fluorescence imaging and immunofluorescence

Live-cell fluorescence imaging was performed using an ECHO Revolve microscope with a 20× objective. To assess detergent sensitivity, cells were first imaged under baseline conditions. Triton X-100 (5% stock in 1× DPBS) was then added directly to the well to achieve a final concentration of 0.5%, followed by a 1-min incubation. The same field of view was subsequently re-imaged to assess detergent-resistant fluorescence.

For immunocytochemistry of p62, cells were fixed at room temperature for 10 min with 4% paraformaldehyde (Electron Microscopy Sciences, 15710) diluted in 1× DPBS, then briefly rinsed with 1× DPBS. Cells were permeabilized and blocked for 10 min in blocking solution consisting of 1×DPBS with 0.1% Triton X-100 and 5% normal goat serum. Primary antibody against p62 (clone D5L7G, Cell Signaling, 88588) was diluted 1:1000 in blocking solution and incubated overnight at 4 °C. The next day, cells were washed three times with 1× DPBS and incubated with a secondary antibody, goat anti-mouse Alexa Fluor 647 (ThermoFisher, A32728). Nuclei were counterstained with Hoechst 33342 (ThermoFisher, 5553141) at a 1:2000 dilution.

For immunostaining of mouse brain tissue, the right hemispheres were drop-fixed in 4% paraformaldehyde overnight at 4°C, then cryoprotected in 30% sucrose prepared in 1× DPBS for at least 24 h. Brains were sectioned at 40 μm thickness, and free-floating sections were blocked in 1× DPBS containing 0.3% Triton X-100 and 5% normal goat serum for 1 h at room temperature. Sections were then incubated overnight at 4°C with a rodent-specific anti-p62 antibody (clone D6M5X, Cell Signaling, 23214) at a 1:500 dilution in blocking buffer. The next day, slices were washed three times for 10 min each in 1× DPBS and incubated with goat anti-rabbit Alexa Fluor 488 secondary antibody (ThermoFisher, A11008) for 1 h at room temperature. Nuclei were counterstained with Hoechst 33342 (1:2000 dilution in 1× DPBS) for 5 min, followed by three additional 10-min washes in fresh 1× DPBS. Sections were then mounted onto microscope slides (Fisher Scientific, 12–550-143) and coverslipped using ProLong Gold antifade mounting medium (Invitrogen, P36930).

Fluorescent images were all immunostained cells and tissues were acquired on the ECHO Revolve on a 20× objective.

### Primary CRISPRi screen and analysis

For screening with the chaperone pooled sgRNA library, 15 million cells containing the FRET reporter were transduced with 1ml of lentivirus and plated on a T175 flask (Day 0). On Day 2, with ~30% of the cells showing BFP positivity, the cells were passaged and replated with 2μg/ml puromycin (Gibco, A1113803). This was repeated on day 5. On day 7, with > 70% of cells showing BFP positivity, the cells were passaged and 20 million cells were plated without puromycin and with the addition of 2 ng/ml doxycycline. The cells were regularly passaged until day 5. The cells were dissociated with trypsin, resuspended in complete DMEM, and sorted using the BD FACSAria Fusion Cell Sorter into FRET-low (~6 million cells) and FRET-high (~2 million cells). Sorting and gDNA isolation for the polyQ chaperone screen was performed twice (two separate times from the same starting population of library-transduced cells). Sorting for the polyG chaperone screen was performed once. Genomic DNA was isolated using a Monarch gDNA extraction kit according to manufacturer protocols. sgRNA-encoding regions were then amplified, followed by sequencing of the protospacers by Illumina NextSeq2000 as recently described [22].

The generation of knockdown phenotypes, p-values, and gene scores to identify the hits used bioinformatics pipelines that we recently described [22], including ‘sgcount’ (https://github.com/noamteyssier/sgcount) and ‘crispr_screen’ (https://github.com/noamteyssier/crispr_screen/). In brief, raw sequencing reads were aligned to a custom reference file containing the CRISPRi chaperone library protospacer sequences using ‘sgcount’, generating sgRNA count matrices for each sample. Using ‘crispr_screen’, these counts were then normalized, and p-values were calculated for individual sgRNAs based on differential abundance between FRET-low and FRET-high. Gene-level phenotypes, including knockdown phenotypes, p-values, and false discovery rates, were derived using the Robust Rank Aggregation (RRA) algorithm [23]. The sgRNA counts matrices and gene-level phenotypes are provided in Supplementary Table 2.

### Secondary assays based on flow cytometry

NLS-FRET-Q79 or NLS-FRET-G100 cell lines were seeded (300,000 cells/well) into 6-well format and transduced with lentivirus packaged from sgRNA-containing pMK1334 packaged. After at least 72 h, reporter expression was induced by doxycycline and analyzed after 5 days by flow cytometry using a BD FACS Fortessa, specifically examining BFP^+^ (sgRNA-containing) cells. Biological replicates of the experiments were from passaging the transduced cells. Gating strategies for representative experiments are shown in Fig. S1. For assessing detergent-resistant aggregates by flow cytometry, we first acquired 10,000 events to determine baseline fluorescence intensity. We then added 5% Triton X-100 diluted in 1× DPBS directly into the tube to a final concentration of 0.5% Triton X-100, gently swirled the tube, and performed flow cytometry to collect another 10,000 events.

Detergent-resistant GFP^+^ aggregates in the eGFP-HTTex1-Q72 cell line shown in Fig. 4 °C were evaluated by flow cytometry after 7 days of doxycycline treatment. A representative gating strategy is shown in Fig. S2. The relative fold-change in fraction of FRET-high cells was obtained by normalizing to sgRNA NTC#1 condition for each experiment.

To test the effect of BFP-DNAJC7 overexpression on GFP-HTTex1-Q72 aggregates, One day after seeding the HEK293T cells (1 × 10^5^ cells/well in 24-well plate), 500 ng of EF1⍺-driven BFP or BFP-tagged DNAJC7 and 250 ng of GFP-HTTex1-Q72 or only 250 ng of GFP-HTTex1-Q72 were diluted into 50 μL of Opti-MEM containing 2.25 μg of PEI . After a 15 min incubation, the mixture was placed dropwise over cells of one well. After 48h, we obtained fluorescence micrographs, followed by dissociating the cells for flow cytometry. We used pulse shape analysis (plotting GFP-Height versus GFP-Width) to quantify aggregates.

### Transfecting cell or tissue homogenates for seeding aggregation

NLS-FRET-Q79 cells containing 2 ng/ml doxycycline were grown in a 10 cm plate for 5 days. The cells were dissociated with trypsin, spun down at 200 g × 10 min, and resuspended in 200 μL 1× DPBS. For tissue homogenates, we used fresh frozen left hemispheres of 22-week-old R6/1 male transgenic mice or age-matched non-transgenic mice. All mice are homozygous for conditional CRISPRi machinery. The right hemispheres of the same mice were drop-fixed in 4% PFA, cryoprotected, and immunostained as described above. The left hemispheres were frozen directly in dry ice and stored at −80 °C. A portion of the fresh-frozen cortex was placed in 200 μL 1×DPBS and homogenized in a 1.5 ml microfuge tube. The crude homogenates from cells or tissues were briefly sonicated using probe (amplitude 10 for 10s), and centrifuged at 20,000g × 15 min at 4 °C, followed by collection of the supernatant into a new tube. The concentrations of the homogenates were estimated by Nanodrop absorbance at 280 nm, using 1 Abs = 1mg/ml. 100 μg of homogenates were diluted into 50 μL Opti-MEM containing 2 μL PEI (1mg/ml), and dispensed dropwise over NLS-FRET-Q79 cells plated in a 24-well format that have been treated with 2 ng/ml doxycycline for one day.

### Immunoprecipitation and western blot

To evaluate DNAJC7 knockdown, NLS-FRET-Q79 cells were transduced with lentivirus containing sgRNAs targeting DNAJC7 or non-targeting controls for 48 h. The were selected for sgRNA-expressing cells using 2 μg/ml puromycin for 3 days, followed by an additional two days of recovery. At 7 days after initial transduction, cells were plated in a 12-well format to confluency. At day 7 of transduction, the cells were lysed with 100 μL of RIPA with protease inhibitors, sonicated briefly, and centrifuged at 21,000 × g for 15 min. The supernatant was collected and combined with LDS buffer (Invitrogen, B0007) and 50 mM DTT (Cell Signaling, 7016. 3 μL of the protein lysate was resolved by SDS-PAGE and western blot as above, with immunoblotting for DNAJC7 and GAPDH (Proteintech, 60004–1-Ig) 1:2,000.

1 × 10^6^ HEK293T cells were plated in a 6-well format and transfected as above with 2 μg 3×FLAG-HTTex1-Q25 or 3×FLAG-HTTex1-Q72 or 3×FLAG-tagged-G100. 48 h after transfection, the cells were lysed with 500 μL of cold IP lysis buffer (20 mM HEPES pH 7.4, 150 mM NaCl, 0.2% IGEPAL CA-630) containing protease inhibitors (Millipore Sigma, 11836170001) and phosphatase inhibitors (Millipore Sigma, 4906845001). The lysates were transferred to a 1.5 ml tube, sonicated briefly, and centrifuged at 21,000g for 20 min. 10 μL of the clarified lysate was taken for input. The remainder of the lysate was incubated with 25 μL of anti-DYKDDDK magnetic agarose beads (Thermo Scientific, A36797) previously washed and equilibrated in IP lysis buffer. The lysates were rotated at 4°C for 2 h, washed three times with 1ml IP lysis buffer, and eluted by addition of 50 μL of 1x LDS buffer shaking at 70 °C for 10 min. After separation from the beads, DTT was added to a final concentration of 50 mM to each sample, boiled for 5 min. Input lysates were also boiled in LDS buffer with 50 mM DTT for 5 min.

5 μL of input lysate and 15 μL of immunoprecipitated eluates were resolved on a 4–20% Tris-Glycine gel (BioRad, 4561096), transferred to a 0.2 μm nitrocellulose membrane (BioRad, 1620112) using a Trans-blot Turbo, and blocked for 1 h with Intercept^®^ Blocking Buffer (LicorBio) before incubated with primary antibody at 4°C overnight in the same buffer. The next day, secondary antibody was incubated for 1h room temperature. Membranes were washed 3× 5 min with 1x TBS with 0.1% Tween after primary antibody and secondary antibody incubation. Primary antibodies include those against: FLAG M2 clone (Millipore Sigma, F3165) 1:5000, DNAJC7 (Proteintech, 11090–1-AP) 1:2000, and GAPDH (Proteintech, 10494) 1:10,000. Secondary antibodies are IRDye^®^ 800CW Goat anti-Rabbit (LicorBio, 926–32211) and IRDye^®^ 700CW Goat anti-Mouse (LicorBio, 926–68070). Membranes were imaged on the Licor Odyssey, and band densities were quantified using ImageJ. Immunoblotting for GAPDH was performed only after imaging FLAG and DNAJC7, and without stripping of the membrane.

### Graphing and statistical analysis

All bar graphs, scatter plots, and volcano plots were generated using R. Statistical tests were performed in R as indicated in the figure legends, except in CRISPR screening analysis which is described separately.

## Results

### A FRET-based reporter of polyQ protein aggregation in the nucleus

**Fig. 1.**
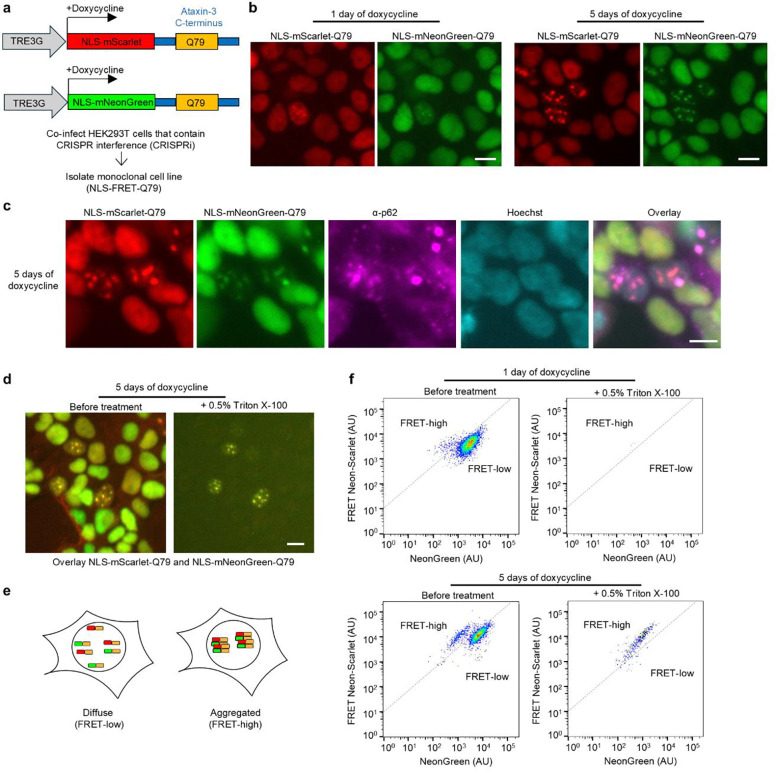
An inducible cell-based model of nuclear, detergent-resistant, p62-positive polyglutamine (polyQ) protein aggregates monitored by a FRET-based reporter. **a**, Schematic of lentiviral constructs for doxycycline-inducible expression of nuclear-localized fluorescent proteins fused to a C-terminal ataxin-3 with 79 glutamines. HEK293T cells engineered with CRISPRi machinery were transduced with these constructs, and clones expressing both fluorescent proteins were selected to generate the NLS-FRET-Q79 cell line. **b**, Fluorescence imaging of NLS-FRET-Q79 cells at one day and five days of doxycycline **c**, Immunofluorescence staining for endogenous p62 in NLS-FRET-Q79 cells after 5 days of doxycycline. **d**, Fluorescence imaging of NLS-FRET-Q79 cells at 5 days of doxycycline before and after treatment with detergent, in the same field of view. **e**, Schematic illustrating how polyQ aggregation gives rise to a “FRET-high” population observable by flow cytometry. **f**, Flow cytometry plots at one day or five days of doxycycline, before and after treatment with detergent. All scale bars are 10 μm.

A common neuropathological hallmark of polyQ diseases is the accumulation of mutant proteins in neuronal nuclei, where they form detergent-resistant, p62-positive inclusions. To model polyQ aggregation, we used the C-terminal segment of ataxin-3, which harbors the polyQ stretch when expanded causes SCA3 and with prior work showing ataxin-3 C-terminal fragments to be highly aggregation prone [24–28]. To model this in a system amenable to high-throughput analysis, we developed a FRET-based reporter system, which has previously proven useful for detecting aggregation of different disease-associated proteins by flow cytometry [29–32]. We selected the highly efficient FRET pair mNeonGreen and mScarlet [33]. HEK293T cells stably expressing CRISPR interference (CRISPRi) machinery were transduced with lentiviral constructs encoding either mNeonGreen or mScarlet, each N-terminally fused to a C-terminal ataxin-3 containing 79 glutamines (Q79), under the control of a doxycycline-inducible Tet-On 3G promoter (Fig. 1a). To specifically target aggregation to the nucleus, we incorporated a strong nuclear localization signal (NLS) into both constructs. Following co-transduction and flow sorting, we isolated a clonal cell line expressing both fluorophores, which we refer to as the NLS-FRET-Q79 reporter line (Fig. 1a).

In the absence of doxycycline, no expression of fluorescent proteins was detected in the NLS-FRET-Q79 cells. One day after doxycycline induction, most cells exhibited diffuse nuclear fluorescence, while a subset displayed small nuclear puncta (Fig. 1b). By five days of induction, these puncta became more prominent and frequent. The puncta contained both mNeonGreen and mScarlet signals, consistent with polyQ-dependent nucleation and co-aggregation. Immunofluorescence staining revealed that the nuclear puncta co-localized with endogenous p62/SQSTM1 (Fig. 1c), a hallmark of polyQ inclusions in human disease. Furthermore, treatment of live cells with 0.5% Triton X-100 resulted in the complete loss of diffuse fluorescence, while the nuclear puncta remained intact (Fig. 1d), indicating that these aggregates are detergent-resistant. We did not observe any evident toxicity from inducing expression of the FRET reporter (not shown).

Aggregation of mNeonGreen and mScarlet is predicted to increase FRET efficiency due to their proximity within polyQ aggregates (Fig. 1e). After one day of doxycycline induction, flow cytometry analysis of FRET intensity versus donor mNeonGreen signal shows a dominant single cluster of cells with a small emerging population with increased FRET signal, but nearly all fluorescence was lost upon detergent-treatment (Fig. 1f). In contrast, after five days of doxycycline induction, a more distinct population of cells emerged with even higher FRET signal that persisted even after detergent treatment, consistent with the presence of polyQ aggregates seen by microscopy at this timepoint. We refer to these two populations as “FRET-high” and “FRET-low,” corresponding to aggregated and non-aggregated states, respectively. Collectively, these data demonstrate that the NLS-FRET-Q79 cell line recapitulates key features of nuclear polyQ aggregation observed in human disease and provides a robust platform for high-throughput detection of aggregate-containing cells by flow cytometry.

### Exogenous polyQ aggregates from HD mice seed the NLS-FRET-Q79 reporter line

**Fig. 2.**
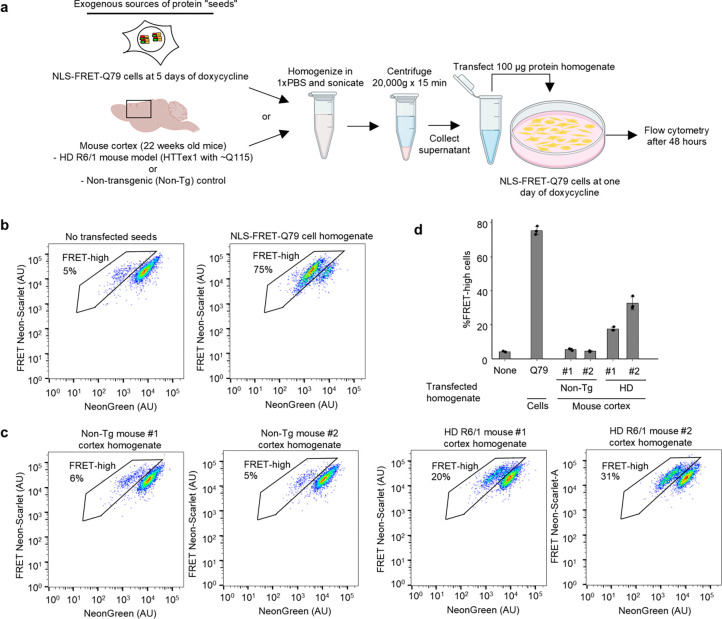
Exogenous polyQ proteins seed aggregation of the polyQ FRET reporter. **a**, Workflow for generating homogenates from cultured cells or mouse cortical tissue, followed by transfection into the NLS-FRET-Q79 reporter cell line. Created in Biorender. **b-d**, Flow cytometry plots showing fraction of FRET-high cells after transfection with homogenates derived from NLS-FRET-Q79 cells (**b**) and cortical homogenates from Huntington’s disease (HD) transgenic mice versus non-transgenic controls (**c**), with quantification of technical triplicates (**d**); bars and error bars represent mean ± standard deviation (sd).

Given the observation that two distinct fluorescent proteins co-aggregate in the NLS-FRET-Q79 cell line, we next sought to determine whether this model reflects general polyQ-dependent protein aggregation. Specifically, we tested whether polyQ aggregates from an external source could seed aggregation of the FRET reporter. To do this, we transfected NLS-FRET-Q79 cells at one day after doxycycline induction with homogenates prepared either from NLS-FRET-Q79 cells treated with doxycycline for five days (as a positive control) or from cortical tissue of transgenic HD mice (Fig. 2a). We used the R6/1 HD mouse model, which expresses exon 1 of human huntingtin (HTTex1) with approximately 115 glutamines. This model exhibits widespread aggregation of mutant HTT throughout the brain, including the cortex, beginning as early as 8 weeks of age and accumulates with age [34,35]. We used cortical tissue from two 22-week-old male R6/1 mice and two age-matched wild-type male mice. To confirm the presence of aggregates in the HD mice, we performed immunostaining on the contralateral hemispheres and detected frequent p62-positive nuclear inclusions in the cortex (Fig. S3), consistent with established pathology in this model.

Transfection with homogenates from NLS-FRET-Q79 cells treated with doxycycline for five days resulted in a robust increase in the FRET-high population, confirming that aggregates generated in this system can seed and propagate further aggregation within the same cell line (Fig. 2b). Notably, transfection with cortical homogenates from R6/1 HD mice led to a strong increase in FRET-high cells compared to homogenates from age-matched control mice (Fig. 2c, d). These results demonstrate that polyQ aggregates derived from HD mouse brain can induce aggregation in the NLS-FRET-Q79 reporter line, suggesting that the pathogenic conformation of polyQ aggregates is conserved between this HD model and the NLS-FRET-Q79 model. These findings also support the NLS-FRET-Q79 reporter as a potentially generalizable model for studying aggregation across polyQ diseases.

### A CRISPRi screen identifies DNAJC7 as a suppressor of polyQ aggregation

**Fig. 3.**
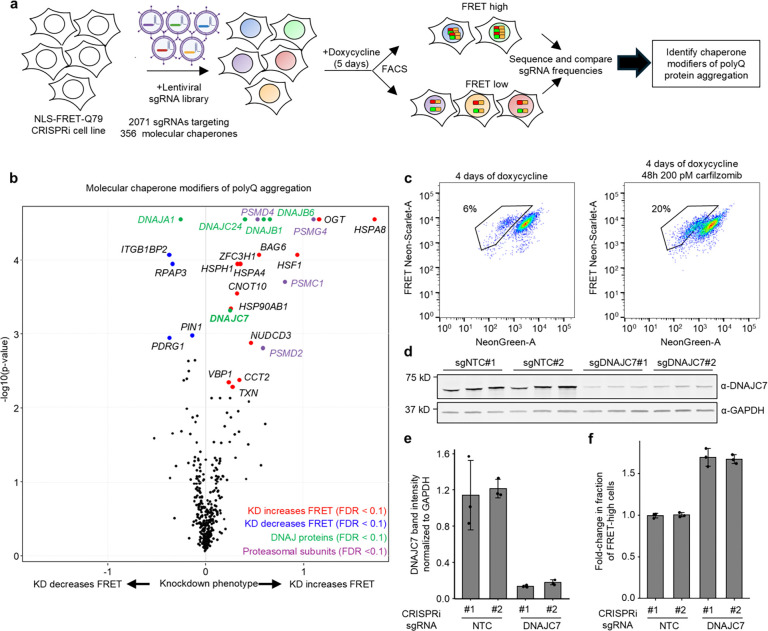
A CRISPRi screen of molecular chaperones identifies DNAJC7 as a modifier of polyQ aggregation. **a**, Workflow of CRISPRi screen to identify molecular chaperone modifiers of polyQ aggregation in NLS-FRET-Q79 cells. Created with BioRender. **b**, Volcano plot CRISPRi screen results, highlighting DNAJ protein and proteasomal subunit hits. **c**, Flow cytometry plots of NLS-FRET-Q79 cells at 4 days of doxycycline with or without proteasomal inhibitor (carfilzomib) **d-e**, Western blot (**d**) and quantification (**e**) of DNAJC7 levels in NLS-FRET-Q79 cells expressing non-targeting (NTC) sgRNAs or sgRNAs targeting DNAJC7; each lane is lysates from separate wells. **f**, Results of flow cytometry measuring fold-change in the fraction of FRET-high cells after 5 days of doxycycline in sgRNA^+^ cells transduced with NTC or DNAJC7. Bars and error bars represent mean ± sd for *n* = 3 independent experiments.

To identify molecular chaperones that modulate polyQ aggregation, we performed a CRISPRi screen in the NLS-FRET-Q79 cell line using a library of 2,103 sgRNAs targeting 356 genes encoding all known molecular chaperones and co-chaperones (screen workflow shown in Fig. 3a). The screen revealed several candidate modifiers of aggregation, including Hsp70 chaperones, members of Hsp40/DNAJ family of co-chaperones, and several proteasomal subunits (Fig. 3b). The top hit whose knockdown increased FRET signal was *HSPA8*, a constitutively expressed and broadly acting Hsp70 family member involved in ATP-dependent protein folding. In addition, several subunits of the proteasome, including *PSMG4*, *PSMD4*, *PSMC1*, and *PSMD2*, were among the top hits. To validate this finding, we treated cells with the proteasome inhibitor carfilzomib, which led to a significant increase in the FRET-high population (Fig. 3c).

Whereas Hsp70s and the proteasome play broad, non-specific roles in protein quality control, the diverse Hsp40/DNAJ family of nearly fifty co-chaperones is thought to confer substrate specificity to Hsp70-mediated refolding [36,37]. Among the top DNAJ family hits in our screen that increased polyQ aggregation were *DNAJB6*, *DNAJB1*, *DNAJC24*, and *DNAJC7*, while knockdown of *DNAJA1* decreased aggregation. These findings are consistent with prior literature: DNAJB6 and DNAJB1 are well-established suppressors of polyQ aggregation [7,8,38–40], and DNAJA1 knockout cells demonstrate reduced polyQ aggregation [41]. Thus, our screen independently recovers known modifiers of polyQ aggregation, validating the robustness of the approach.

Importantly, in addition to known suppressors, the screen identified *DNAJC7* and *DNAJC24* as previously unexplored hits that significantly increased polyQ aggregation when knocked down. Between these two genes, we prioritized *DNAJC7* for further investigation based on several lines of evidence. Firstly, it is highly expressed in the brain [42]. Secondly, it was recently reported to interact with or modify aggregation of other neurodegenerative disease-associated proteins, including Tau and TDP-43 [43–46]. Thirdly, loss-of-function mutations in *DNAJC7* have been linked to amyotrophic lateral sclerosis [47,48]. Finally, it has been previously identified within polyQ inclusions in mouse neuroblastoma cell models [49,50]. In contrast, while DNAJC24 was a stronger hit, it has low brain expression and no known links to neurological disease.

To validate the effect of *DNAJC7* knockdown, we generated NLS-FRET-Q79 lines stably expressing either two different non-targeting control sgRNAs or sgRNAs targeting *DNAJC7*. Western blotting confirmed efficient knockdown of DNAJC7 protein (Fig. 3d, e). Importantly, flow cytometry showed a significant increase in the fraction of FRET-high cells upon *DNAJC7* knockdown (Fig. 3f). These findings confirmed that DNAJC7 is an endogenous suppressor of polyQ aggregation in the context of this FRET reporter system.

### DNAJC7 physically interacts with mutant HTT exon 1 in cells and suppresses aggregation

**Fig. 4.**
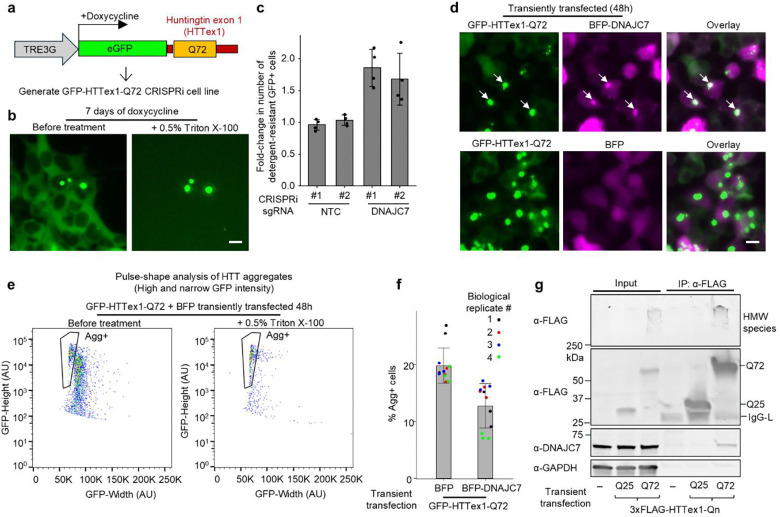
DNAJC7 physically interacts with mutant HTT exon 1 and suppresses its aggregation. **a**, Schematic of a lentiviral construct encoding a doxycycline-inducible eGFP-tagged fragment of Huntingtin exon 1 containing 72 glutamines (GFP-HTTex1-Q72), used to generate a monoclonal HEK293T CRISPRi cell line. **b**, Fluorescence micrograph of GFP-HTTex1-Q72 cells at 7 days of doxycycline treatment before and after treatment with Triton X-100. **c**, Results of flow cytometry experiments measuring increases the fraction of total events containing detergent-resistant GFP^+^ cells comparing those stably transduced with sgRNAs that are NTCs or targeting DNAJC7. Data represent mean ± sd from *n* = 4 independent experiments. **d**, Fluorescence micrographs of HEK293T cells 48h after transient co-transfected GFP-HTTex1-Q72 and either mTagBFP2 (BFP) alone or BFP-tagged DNAJC7. Arrows indicate cytoplasmic aggregates. **e**, Flow cytometry plotting GFP-height versus GFP-Width (pulse shape analysis) of HEK293T cells transfected with GFP-HTTex1-Q72 for 48h high. The detergent-resistant population of cells are designated as aggregate-positive (Agg^+^). **f**, Results of flow cytometry experiments measuring Agg^+^ cells in HEK293T cells 48h after transient co-transfection with GFP-HTTex1-Q72 and either BFP or BFP-DNAJC7. Bars and error bars represent mean ± sd from *n* = 4 independent biological replicates (indicated by color), with each performed in technical triplicate. **g**, Western blot of input lysates and α-FLAG immunoprecipitated lysates from HEK293T cells transfected with 3×FLAG-tagged HTTex1 with either 25 or 72 glutamines and immunoblotted for FLAG, DNAJC7, and GAPDH. HMW: High molecular weight. IgG-L: Immunoglobulin light-chain. All scale bars are 10 μm.

To determine whether DNAJC7 modifies aggregation in other polyQ disease contexts, and to exclude the possibility that its effects are specific to the nucleus or the specific FRET pair fluorescent proteins, we generated an independent reporter line. Specifically, we developed a monoclonal HEK293Ti CRISPRi cell line expressing doxycycline-inducible eGFP-tagged huntingtin exon 1 containing 72 glutamines (GFP-HTTex1-Q72) (Fig. 4a). Unlike the NLS-FRET-Q79 construct, HTTex1 naturally contains an N-terminal nuclear export signal, resulting in primarily cytoplasmic aggregation in HEK293T cells [51,52]. After seven days of doxycycline induction, we observed detergent-resistant cytoplasmic GFP+ inclusions in a subset of cells (Fig. 4b). CRISPRi knockdown of *DNAJC7* in the GFP-HTTex1-Q72 cell line significantly increased the fraction of detergent-insoluble GFP+ aggregates, as measured by flow cytometry (Fig. 4c), indicating that DNAJC7 suppresses aggregation in the cytoplasm for a different polyQ protein context.

We next sought to determine whether DNAJC7 co-localizes with HTTex1 aggregates. However, attempts to visualize endogenous DNAJC7 by immunostaining were unsuccessful due to the lack of a suitable antibody. To address this, we performed transient co-transfection experiments in HEK293T cells using GFP-HTTex1-Q72 together with either mTagBFP2 (BFP) alone or BFP-tagged DNAJC7. As expected, GFP-HTTex1-Q72 formed cytoplasmic aggregates. Notably, BFP-DNAJC7 co-localized with a subset of these aggregates, whereas BFP alone did not (Fig. 4d), suggesting an interaction between DNAJC7 and aggregated HTTex1.

To assess whether DNAJC7 overexpression could reduce HTTex1 aggregation, we used flow cytometry pulse-shape analysis, which distinguishes aggregates based on a characteristic high-intensity and narrow-width fluorescence signal [40,53]. At 48 h post-transfection, coinciding with the appearance of aggregates by microscopy, we observed a distinct population of cells with high and narrow GFP signal, designated as Agg⁺ (aggregate-positive), which persisted following detergent treatment (Fig. 4e). Compared to BFP control, overexpression of BFP-DNAJC7 significantly reduced the fraction of Agg⁺ cells (Fig. 4f), indicating that DNAJC7 suppresses aggregation of mutant HTTex1 when overexpressed. BFP-DNAJC7 did not reduce the total number of GFP-positive cells compared to BFP (Fig. S4), indicating similar transfection efficiencies.

To further establish whether endogenous DNAJC7 interacts with mutant HTT, we performed anti-FLAG immunoprecipitation from HEK293T cells expressing 3×FLAG-tagged HTTex1 with either 25 or 72 glutamines. We confirmed that endogenous DNAJC7 co-immunoprecipitated to a much greater degree with Q72 construct than with Q25 or lysates of non-transfected cells (Fig. 4g). (Biological replicates of this finding are described later). We also observed the presence of high molecular weight (HMW) species at the top of the gel in the Q72 condition, confirming HTTex1-Q72 aggregation.

### A FRET-based reporter for polyG aggregation reveals detergent-resistant, p62-positive nuclear inclusions and seeding activity

Given the emerging role of DNAJC7 as a modifier of multiple disease-associated protein aggregates, we next sought to examine its relevance in a distinct repeat expansion disorder. We focused on neuronal intranuclear inclusion disease (NIID), which is caused by an expanded polyG tract encoded within the upstream open reading frame of the *NOTCH2NLC* (uN2C) gene and, much like the polyQ diseases, is pathologically characterized by the presence of p62-positive nuclear inclusions [15,54].

**Fig. 5.**
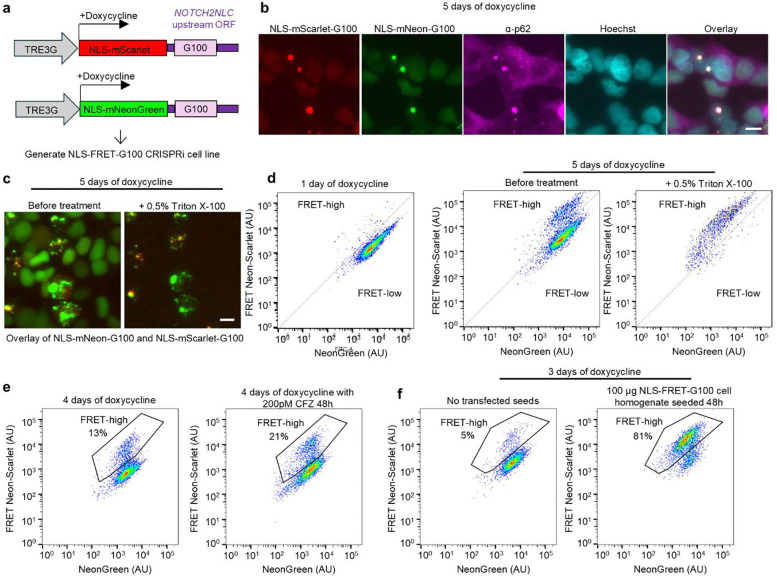
An inducible model of polyglycine (polyG) aggregation reveals detergent-resistant, p62-positive nuclear inclusions and seeding activity. **a**, Lentiviral constructs for generating NLS-FRET-G100 cell line expressing the upstream open reading frame (uORF) of the *NOTCH2NLC* gene with polyglycine tract of 100 residues. **b**, Immunofluorescence staining for p62 of NLS-FRET-G100 cells at 5 days of doxycycline. **c**, Fluorescence imaging of NLS-FRET-Q79 cells at 5 days of doxycycline before and after detergent treatment, in the same field of view. **d**, Flow cytometry plots at one and five days of doxycycline treatment, and the latter before and after detergent treatment. **e**, Flow cytometry plots of NLS-FRET-Q79 cells at 4 days of doxycycline with or without proteasomal inhibitor (carfilzomib). **f**, Flow cytometry results of NLS-FRET-G100 cells at 3 days of doxycycline with and without transfection with NLS-FRET-G100 cell homogenates (collected five days after doxycycline) for 48h. All scale bars are 10 μm.

Using a parallel approach to our polyQ model, we developed a clonal HEK293T CRISPRi cell line containing a doxycycline-inducible, nuclear-localized FRET reporter expressing uN2C containing 100 glycines (designated NLS-FRET-G100) (Fig. 5a). Five days after doxycycline induction, a subset of cells exhibited nuclear puncta positive for both fluorophores, which co-localized with p62 (Fig. 5b). These puncta were resistant to Triton X-100 treatment, indicating that they form detergent-insoluble aggregates (Fig. 5c).

Flow cytometry revealed a progressive increase in the FRET-high population over time, which was maintained after detergent treatment (Fig. 5d). Inhibition of the proteasome with carfilzomib further increased the FRET-high population (Fig. 5e). Finally, we found that transfection of homogenates derived from NLS-FRET-G100 cells into accelerate aggregation of the reporter in living cells (Fig. 5f), confirming that polyG aggregates possess seeding activity. Notably, these same homogenates did not induce aggregation in the NLS-FRET-Q79 line (Fig. S5), suggesting that polyG and polyQ aggregates are biochemically distinct.

Together, these findings establish NLS-FRET-G100 as a robust live-cell reporter of nuclear polyG aggregation that recapitulates key pathological features of NIID and enables quantitative, high-throughput analysis.

### DNAJC7 partially physically interacts with but does not suppress polyG aggregation

**Fig. 6.**
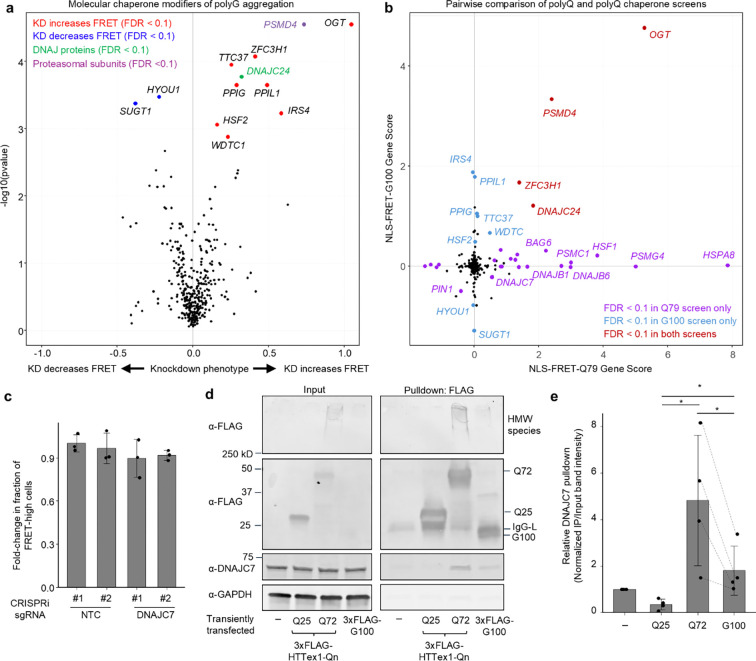
DNAJC7 partially physically interacts with polyG but does not alter its aggregation. **a**, Volcano plot showing results of a CRISPRi screen for molecular chaperone modifiers of polyG aggregation in the NLS-FRET-G100 cell line. **b**, Pairwise comparison of Gene Scores between the polyG screen (from panel **a**) and the polyQ screen (Fig. 3b). **c**, Results of flow cytometry experiments measuring fold-change in the fraction of FRET-high cells after 5 days of doxycycline in sgRNA^+^ cells transduced with NTC or DNAJC7. Data represent mean ± sd from *n* = 3 independent experiments. **d**, DNAJC7 co-immunoprecipitates with 3xFLAG-tagged G100 to a greater extent than with untransfected lysates or HTTex1-Q25, but to a lesser extent than with HTTex1-Q72, in transiently transfected HEK293T cells. HMW: High molecular weight. IgG-L: Immunoglobulin light-chain **e**, Quantification of DNAJC7 co-immunoprecipitation from *n* = 4 independent experiments. Dotted lines connect paired values for Q72 and G100 from the same immunoblot. Bars and error bars represent mean ± sd. *p<0.05 by paired *t*-test.

Using the same CRISPRi screening approach as with the NLS-FRET-Q79 cell line, we performed a molecular chaperone screen in the NLS-FRET-G100 reporter line to identify modifiers of polyG aggregation (Fig. 6a). Surprisingly, this screen revealed relatively few significant hits. Notably, key polyQ modifiers such as *DNAJC7*, *DNAJB6*, *DNAJB1*, and *HSPA8* were not identified as hits in the polyG screen. A direct comparison of Gene Scores between the polyQ and polyG screens revealed minimal overlap in chaperone modifiers (Fig. 6b), suggesting distinct mechanisms of proteostasis regulation between polyQ and polyG aggregates.

Among the few shared hits was *OGT*, which encodes O-GlcNAc transferase. *OGT* knockdown was among the top hits in both screens, and we independently validated that its depletion significantly increases the fraction of FRET-high cells in both the NLS-FRET-Q79 and NLS-FRET-G100 lines (Fig. S6), confirming the reliability of the polyG screen. In contrast, direct knockdown of *DNAJC7* had no significant effect on the proportion of FRET-high cells in the NLS-FRET-G100 model (Fig. 6c), further supporting a differential requirement for DNAJC7 in regulating polyQ versus polyG aggregation, at least as detected by FRET.

Despite the lack of a functional effect of *DNAJC7* knockdown on polyG aggregation, co-immunoprecipitation experiments revealed that DNAJC7 still partially interacts with polyG proteins. In transiently transfected HEK293T cells, endogenous DNAJC7 co-immunoprecipitated with FLAG-tagged G100 to a greater extent than with HTTex1-Q25 or untransfected controls, but consistently to a lesser extent than with HTTex1-Q72 (Fig. 6d, e). This suggests that while DNAJC7 can interact with polyG-expanded protein, the interaction is either weaker or is with a small subset of microscopically visible polyG aggregates. There was also a small amount of HMW species detected in the G100 condition and to a lesser degree than seen with HTTex1-Q72.

## Discussion

In this study, we developed new inducible FRET-based reporter systems for polyQ and polyG aggregation that recapitulate features of human disease and enable dynamic monitoring of aggregation. Importantly, these reporters allow for high-throughput flow cytometry-based genetic screens, which we used to systematically test all known molecular chaperones to identify modifiers of aggregation. Beyond screening applications, these models also provide a valuable platform for exploring the relatively understudied nuclear proteostasis pathways, and they could serve as biosensors to assess seeding activity of mouse or human brain homogenates or other biospecimens.

By taking a comprehensive approach, we were able to identify for the first time the Hsp40 co-chaperone DNAJC7 as a potent modifier of polyQ protein aggregation. A major next step is to understand the functional impact of DNAJC7 in the central nervous system (CNS), particularly given its high expression among Hsp40 family members. DNAJC7 appears to be especially important in motor neurons, supported by its genetic link to amyotrophic lateral sclerosis (ALS) and recent evidence that loss of DNAJC7 in iPSC-derived motor neurons increases susceptibility to proteotoxic stress, in part due to impaired HSF1 signaling [55]. However, its role may extend more broadly across the CNS. Prior studies have shown that DNAJC7 interacts with other aggregation-prone proteins, including Tau and TDP-43, with Hou et al. demonstrating that mutant tau co-precipitates with anti-DNAJC7 antibodies in a transgenic mouse model of frontotemporal dementia[44]. These findings suggest that DNAJC7 may act as a general regulator of protein aggregation in neurodegenerative disease. Our study was limited by the availability of an antibody that could reliably immunostain DNAJC7 to test its colocalization in mouse or human brain tissues to further validate this finding.

A central question raised by our study, and by the growing number of reported DNAJC7 substrates, is the molecular basis of its substrate specificity. While DNAJC7 has been shown to bind and stabilize native tau [44], we did not observe increased interaction with HTTex1-Q25, suggesting that an aggregation-prone polyQ tract is required for recognition. Intriguingly, despite their high aggregation propensity, polyG proteins show only weak interaction with DNAJC7, and knockdown of DNAJC7 did not affect polyG aggregation. One possibility is that polyG aggregates adopt liquid-like or gel-like conformations that enhance FRET but are not competent for recognition by the Hsp40/Hsp70 machinery. As previously demonstrated for DNAJB6, DNAJC7 may preferentially bind to fibrillar or amyloid-like structures, a biophysical state shared by tau and polyQ aggregates [56–58]. To date, the amyloidogenic potential of polyG aggregates remains poorly defined. Future studies using live-cell imaging approaches such as fluorescence recovery after photobleaching (FRAP), along with biochemical characterization, will be valuable for distinguishing the conformational states of polyQ versus polyG aggregates and for understanding how these states differentially engage the cellular chaperone network.

It is also notable that DNAJC7 is structurally distinct among Hsp40 family members, as it contains multiple tetratricopeptide repeat (TPR) domains in addition to the canonical J-domain. Beyond the shared J-domain, DNAJC7 has no significant sequence homology with DNAJB6 or other Hsp40s, suggesting that it may recognize and engage substrates through a different mechanism. Although we observe physical interaction between DNAJC7 and polyQ proteins in cells, it remains unclear whether this reflects direct binding to misfolded species or occurs through intermediary factors. As previously shown with tau [44,59], biochemical reconstitution studies using purified proteins will be critical to determine whether DNAJC7 directly recognizes misfolded polyQ aggregates, and to dissect the specific contribution of its TPR domains to client binding and chaperone activity.

Beyond DNAJC7, our CRISPRi screens uncovered several additional candidate modifiers of protein aggregation that merit further investigation. One of the most prominent was OGT, the enzyme responsible for O-GlcNAcylation, whose knockdown markedly increased aggregation in both the polyQ and polyG models. O-GlcNAcylation is a dynamic post-translational modification that regulates a wide array of proteins and cellular processes, including in the brain [60–63], with our findings suggesting a potential role in proteostasis. Another notable hit specific to polyQ protein aggregates was BAG6, a chaperone “holdase” required for ubiquitin-mediated degradation of newly synthesized misfolded polypeptides has previously been shown to modulate the aggregation of TDP-43 fragments [64–66]. Additionally, hits unique to the polyG screen included members of the peptidyl-prolyl isomerase family, such as *PPIG* and *PPIL1*, which have been implicated in modulating tau aggregation [67]. These findings highlight the potential of our platform to identify both shared and distinct regulators of aggregation across different disease-relevant proteins.

## Conclusions

This work establishes a robust and scalable platform for studying the aggregation of polyQ and polyG proteins, leveraging inducible FRET-based reporters that enable high-throughput genetic screening in live cells. Our CRISPRi screens provide new insights into the molecular chaperone network and its role in regulating aggregation, uncovering both shared and distinct modifiers between two different repeat expansion disorders. Given the scalability of this system, ongoing efforts to extend these screens to a genome-wide level are expected to uncover additional regulators, offering deeper understanding of proteostasis mechanisms and informing the development of targeted therapeutic strategies for neurodegenerative diseases.

## Supplementary Material

Supplement 1

Supplement 2

Supplement 3

Supplement 4

## List of abbreviations

ALS: Amyotrophic lateral sclerosis
CRISPRi: CRISPR interference
FRET: Föster resonance energy transfer
HD: Huntington’s disease
HTTex1: Huntingtin exon 1
NIID: Neuronal intranuclear inclusion disease
NLS: Nuclear localization signal
OGT: O-GlcNAc transferase
PolyG: Polyglycine
PolyQ: Polyglutamine
SCA: Spinocerebellar ataxia
TDP-43: TAR DNA-binding protein 43
TPR: Tetratricopeptide repeat
uN2C: Upstream open reading frame of *NOTCH2NLC*
